# Clinical Trial: Effects of Autologous Dendritic Cell Administration on Renal Hemodynamics and Inflammatory Biomarkers in Diabetic Kidney Disease

**DOI:** 10.3390/diseases13040122

**Published:** 2025-04-21

**Authors:** Endang Drajat, Aziza Ghanie Icksan, Jonny Jonny, Aditya Pratama Lokeswara, Bhimo Aji Hernowo, Elvita Rahmi Daulay, Terawan Agus Putranto

**Affiliations:** 1 Faculty of Medicine, Dentistry, and Health Sciences, Universitas Prima Indonesia, Medan 20118, Indonesia; endangdrajat36@gmail.com (E.D.); jonny@unprimdn.ac.id (J.J.); hernowobhimo@gmail.com (B.A.H.); elvitarahmidaulay@gmail.com (E.R.D.); 2 Department of Radiology, Gatot Soebroto Army Hospital, Jakarta 10410, Indonesia; lokeswaraaditya@gmail.com; 3 Faculty of Medicine, Universitas Pembangunan Nasional “Veteran” Jakarta, Jakarta 12450, Indonesia; 4 Faculty of Military Medicine, Indonesia Defense University, Bogor 16810, Indonesia; 5 Nephrology Division, Department of Internal Medicine, Gatot Soebroto Army Hospital, Jakarta 10410, Indonesia; 6 Indonesia Army Cellcure Center, Gatot Soebroto Army Hospital, Jakarta 10410, Indonesia

**Keywords:** diabetic kidney disease, dendritic cells, cell therapy, doppler ultrasonography, Transforming Growth Factor Beta, Matrix Metalloproteinase-9

## Abstract

Background: Diabetic kidney disease (DKD) is a significant risk factor for End-Stage Renal Disease, with a high global incidence and mortality rate. Hyperglycemia in DKD induces inflammation, contributing to glomerular hyperfiltration, fibrosis, and impaired renal function. Current therapies, including SGLT2 inhibitors, ACE inhibitors, and ARBs, show limited efficacy. Autologous dendritic cells (DCs) offer potential anti-inflammatory effects by reducing cytokine activity and fibrosis biomarkers. Methods: A quasi-experimental pretest–post-test design was conducted involving 29 DKD patients. Baseline blood and urine samples were collected for MMP-9, TGF-β, and Doppler ultrasound (PSV, EDV) measurements. The subjects received subcutaneous injections of autologous DCs, and follow-up measurements were conducted four weeks after treatment. The statistical analyses included paired t-tests, Wilcoxon signed-rank tests, and linear regression. Results: After treatment, there were a significant decrease in PSV (from 47.1 ± 23.87 cm/s to 27.85 ± 20.53 cm/s, *p* = 0.044) and a significant increase in EDV (from 13 ± 5.32 cm/s to 15.7 ± 12.55 cm/s, *p* = 0.039). A strong correlation was observed between the TGF-β and MMP-9 levels (*p* = 0.001). Linear regression analysis showed reduced MMP-9 influence on the TGF-β after treatment, suggesting potential fibrosis reduction. Gender and UACR subgroup analyses revealed significant PSV and EDV improvements in females and the microalbuminuria group. Conclusion: Autologous dendritic cell therapy significantly improved renal hemodynamics and showed potential to reduce fibrosis by modulating TGF-β and MMP-9 levels in DKD patients, warranting further investigation.

## 1. Introduction

Diabetic kidney disease (DKD) is a significant risk factor for the development of End-Stage Renal Disease (ESRD) [1]. Globally, an estimated 2.6 million new cases of DKD are diagnosed annually, and this number is projected to rise further [2]. The mortality rate among individuals with DKD is alarmingly high, reaching 31.1% worldwide [3].

In DKD, hyperglycemia induces a pro-inflammatory state, leading to elevated cytokine production and subsequent renal damage [4]. Inflammation affects renal hemodynamics, causing alterations in blood flow that initially result in glomerular hyperfiltration, a hallmark of early DKD progression [5]. Doppler ultrasound measurements, such as Peak Systolic Velocity (PSV) and End-Diastolic Velocity (EDV), provide non-invasive estimates of intraglomerular pressure, which are pivotal in understanding glomerular hyperfiltration [6]. Chronic inflammation further exacerbates kidney damage through fibrosis, which plays a central role in declining renal function. Studies suggest that the activation of MMP-9 facilitates the conversion of latent TGF-β into its active form, promoting fibrotic processes [7].

Recent discoveries suggest that autologous dendritic cell transfer holds promise as a therapeutic approach to alleviate inflammation [8,9,10]. These cells can be engineered ex vivo to enhance their immune-regulatory properties and subsequently reintroduced into a patient’s body, promoting immune tolerance and mitigating inflammatory responses [11,12]. The therapeutic application of autologous dendritic cells has demonstrated potential in reducing inflammation and fibrosis across various conditions, positioning them as a compelling candidate for managing DKD [13,14]. This study aims to evaluate the effects of autologous dendritic cell administration on renal hemodynamics and fibrosis biomarkers, exploring their potential to address the challenges of DKD treatment.

## 2. Materials and Methods

### 2.1. Study Design

This study utilized a quasi-experimental design using a one-group pretest–post-test approach. This research was conducted at the Army Central Hospital with participants selected through a nonprobability sampling technique. Ethical approval for this study was granted by the Ethics Committee of the Army Hospital (Ethical Clearance No. 109/VIII/KEPK/2024, dated 23 August 2024). All participants provided written informed consent prior to their inclusion in this study. The clinical trial is registered with ClinicalTrials.gov under the trial registration number NCT06866158. The trial was first submitted on 22 February 2025.

### 2.2. Research Subject

A total of 10,930 subjects were initially identified from polyclinics within the hospital, with 1280 subjects from the endocrine clinic and 312 from the renal clinic. Among them, 36 subjects agreed to participate in this study. After applying of the exclusion criteria, 29 subjects completed this study and underwent ultrasound evaluations.

The inclusion criteria were required to meet the diagnostic criteria for Diabetes Mellitus according to the PERKENI 2021 guidelines, be over 18 years of age, provide written informed consent, and have an eGFR of ≥30 mL/min/1.73 m^2^ and a Urinary Albumin-to-Creatinine Ratio (UACR) of ≥ 30 mg/g. Additionally, the subjects needed to demonstrate an understanding of and willingness to comply with study procedures.

The exclusion criteria included receiving immunosuppressive therapy within the past four weeks, a history of other kidney diseases, alternative Diabetes Mellitus diagnoses, a positive pregnancy test, prior thromboembolism or genetic predisposition to thromboembolism, and use of anti-thromboembolic therapies other than low-dose aspirin. Subjects were also excluded if they had physical or mental disabilities preventing daily activities, medical conditions that could interfere with this study (e.g., acute, subacute, intermittent, or chronic conditions posing risk), excessive obesity (BMI > 40), or uncontrolled hypertension (systole > 180 mmHg, diastole > 100 mmHg) or were unwilling to provide written informed consent. These criteria ensured a carefully selected cohort for robust and reliable study outcomes.

### 2.3. Research Procedure

The research procedure began with the preparation of the subjects, which included collecting blood samples for baseline measurements of MMP-9 and TGF-β using sandwich ELISA kits (Reed Biotech Ltd., Wuhan, China) and for the generation of autologous dendritic cells. Additional procedures involved urine collection and Doppler ultrasound assessment of renal blood flow parameters: specifically, Peak Systolic Velocity (PSV) and End-Diastolic Velocity (EDV). The Doppler ultrasound examinations, performed by two specialist radiologists using a Siemens Acuson Sequoia ultrasound machine, focused on the interlobar arteries of the right and left kidneys, with the results averaged for both kidneys.

One week after the initial assessments, the autologous dendritic cells were injected subcutaneously into each subject’s arm. Four weeks following the injection, the cytokine levels (MMP-9 and TGF-β) were reassessed, along with the renal blood flow velocities (PSV and EDV), using the same Doppler ultrasound methodology. This comprehensive approach ensured the accurate and consistent evaluation of both biochemical and hemodynamic changes throughout this study.

### 2.4. Autologous Dendritic Cell Generation

At baseline, 40 cc of blood was drawn from each subject. The blood was processed and incubated in a medium containing GM-CSF (Granulocyte Macrophage Colony Stimulating Factor) and IL-4 for five days to generate the dendritic cells. Subsequently, the antigen was added and incubated for two additional days to induce the maturation of the dendritic cells. Finally, the prepared autologous dendritic cells were administered by subcutaneous injection into each subject’s arm.

### 2.5. Safety Evaluation

The injections were administered by a qualified physician, with the subjects monitored for 30 min after injection to observe any potential allergic reactions. Any adverse events meeting the criteria outlined in CTCAE v6.0 were documented for up to seven days following the injection. This study adhered strictly to both local and international regulations, aligning with the principles of the Declaration of Helsinki.

### 2.6. Statistics

A data normality test was conducted on each variable. The Shapiro–Wilk normality test was used for samples below 50, while the Kolmogorov–Smirnov test was used for samples above 50. The PSV and EDV variables were analyzed using a paired *t*-test for normally distributed data, while the non-normally distributed data were analyzed using the Wilcoxon signed-rank test. The MMP-9 and TGF-β variables used linear regression analysis tests to see the effects before and after the administration of the autologous dendritic cells.

### 2.7. GenAi Disclosure

This manuscript has been reviewed and enhanced with the assistance of a generative AI tool (Grammarly) to improve the language clarity, grammar, and overall readability. The tool was used solely for editorial purposes and does not alter the scientific content or methodology of this study. All decisions regarding the study design, data interpretation, and conclusions remain the responsibility of the authors.

## 3. Results

### 3.1. Subject Characteristics

Table 1 presents the characteristics of the research subjects, comprising 29 participants. The majority of subjects were aged over 60 years. In terms of gender distribution, there were slightly more females (16) than males (13). Hypertension was the most prevalent comorbidity, affecting 96.6% of the participants. Regarding antidiabetic medication use, insulin was the most commonly utilized drug, reported by 69% of subjects. Similarly, angiotensin receptor blockers (ARBs) were the most frequently used antihypertensive medication, with 72.4% of participants reporting their use.

### 3.2. PSV and EDV Results

Table 2 shows the changes in the PSV and EDV. The autologous dendritic cell administration showed significant changes in the PSV and EDV parameters. Before the dendritic cell administration, the median PSV value was 47.1 ± 23.87 cm/s. After the dendritic cell administration, the median PSV value decreased to 27.85 ± 20.53 cm/s. This decrease was statistically significant, with a p-value of 0.044. The median EDV value before the administration was 13 ± 5.32 cm/s. After the dendritic cell administration, the median EDV value decreased to 15.7 ± 12.55 cm/s. This decrease was statistically significant, with a *p*-value of 0.039.

Table 3 shows the PSV values based on gender, age, and UACR. In the male group, the median PSV value before the dendritic cell administration was 47.77 ± 14.96 cm/s, and after the dendritic cell administration, the median value decreased to 27.05 ± 42.38 cm/s, although this was not statistically significant (*p* = 0.422). In the female group, there was a significant decrease from 51.65 ± 24.8 cm/s to 31.72 ± 18.31 cm/s with a *p*-value = 0.03.

In the age group of below 60 years, the mean value of the PSV before the dendritic cell administration was 52.56 ± 18.41 cm/s, and after the dendritic cell administration, the mean value decreased to 42.32 ± 24.80 cm/s, although this was not statistically significant (*p* = 0.225). In the age group of above 59 years, there was a decrease in the median value from 47.02 ± 24.97 cm/s to 29 ± 20.43 cm/s with a *p*-value = 0.121.

Changes in the PSV were also found in the UACR group before and after the administration of the autologous dendritic cells. In the microalbuminuria group, the PSV before the administration of the autologous dendritic cells had a median value of 54.6 ± 23.46 cm/s. The PSV after the autologous dendritic cell administration had a median value of 27.65 ± 16.74 cm/s. Hypothesis testing with a *p*-value of 0.011 showed that this is a significant difference. In the macroalbuminuria group, the median value of the PSV before the administration of the autologous dendritic cells was 47.05 ± 32.3 cm/s. The PSV after the administration of the autologous dendritic cells had a median value of 35.7 ± 32.28 cm/s. Hypothesis testing showed that this change was not significant, with a *p*-value of 0.834.

Table 4 shows the EDV analysis based on gender, age, and UACR group. In the EDV analysis based on gender, the results show that in the male group, the median EDV value before the dendritic cell administration was 12.55 ± 6.97 cm/s. After the dendritic cell administration, there was an increase to 15.7 ± 21.9 cm/s. This increase is not statistically significant, with a *p*-value of 0.249. In the female group, the median EDV value before the dendritic cell administration was 13.27 ± 6.8 cm/s. After the dendritic cell administration, there was a significant increase to 15.04 ± 11.08 cm/s, with a *p*-value of 0.044.

In the age group of below 60 years, the mean value of the EDV before the administration of the autologous dendritic cells was 15.53 ± 6.10 cm/s. After the administration of the autologous dendritic cells, there was an increase in the mean value of the EDV by 23.03 ± 14.93 cm/s. This increase was not statistically significant, with a *p*-value of 0.137. In the age group of above 60 years, the median value before the administration of the autologous dendritic cells was 4.11 ± 6.08 cm/s. After the administration of the autologous dendritic cells, there was an increase in the median value of 12.64 ± 11.08 cm/s. This increase was not statistically significant, with a *p*-value of 0.126.

The EDV in the microalbuminuria group had a median value of 13.8 ± 5.36 cm/s before the autologous dendritic cell administration; after the autologous dendritic cell administration, the median value increased to 14.19 ± 11.18 cm/s. This increase was not statistically significant, with a *p*-value of 0.234. In the macroalbuminuria group, the median value before the administration of the autologous dendritic cells was 11.15 ± 6.28 cm/s. After the administration of the dendritic cells increased to 16.4 ± 17.75 cm/s, this increase was not statistically significant, with a *p*-value of 0.234.

### 3.3. TGF-β and MMP-9 Results

Table 5 shows the linear regression testing of the TGF-β and MMP-9 before and after the administration of the autologous dendritic cells. The linear regression test before the action showed that every increase of one unit of MMP-9 would increase the TGF-β by 13.112, and this result was close to significant, with a *p*-value = 0.058. The linear regression test after the treatment showed that every one unit increase in MMP-9 would increase the TGF-β by 7.622, with a near significant *p*-value (*p*-value = 0.066). However, when comparing the value of the MMP-9 to the TGF-β before and after the autologous dendritic cell administration, there was a decrease in the value of the MMP-9 to that of the TGF-β.

Table 6 shows the analysis of the relationship between the study variables. The variables before and after the administration of the autologous dendritic cells were combined, and then the correlation test between the variables was performed.

The relationship test between the research variables using Spearman showed that there was a significant relationship between the TGF-β and MMP-9, with a *p*-value of 0.001. There was also a significant relationship between the PSV and EDV, with a *p*-value of 0.000.

## 4. Discussion

A significant portion of the study population (93.1%) had a history of hypertension, a known major risk factor for DKD. This aligns with the existing literature, where hypertension is recognized as one of the most significant contributors to the progression of kidney disease [15]. The pathophysiology of hypertension in DKD involves the activation of the vasoactive hormone pathway, leading to glomerular hyperfiltration, increased glomerular pressure, and subsequent inflammation and fibrosis [16].

In this cohort, angiotensin receptor blockers (ARBs) were the most commonly prescribed antihypertensive medication. Research supports the use of ARBs and ACE inhibitors in reducing Urinary Albumin-to-Creatinine Ratios (UACRs) and improving renal function in DKD patients [17]. However, as noted, combining ARBs with mineralocorticoid receptor antagonists can increase the risk of hyperkalemia [18].

This study showed that there were no significant differences between the UACR groups for PSV, EDV, TGF-β, and MMP-9 before the administration of the autologous dendritic cells. In addition, there were no significant differences based on the history of the disease or the use of diabetic drugs or a history of antihypertensive drug use.

Significant changes in the PSV and EDV were observed following the dendritic cell therapy, indicating improvement in renal blood flow and perfusion. PSV and EDV are important hemodynamic indicators of renal artery resistance, with a higher PSV often correlated with increased renal resistance and a pro-inflammatory state. Several studies have shown that increased PSV is associated with elevated levels of C-reactive protein (CRP), a marker of inflammation, especially in hypertensive and diabetic patients [19]. Similarly, a higher PSV typically correlates with a lower GFR, as increased vascular resistance impairs kidney filtration. This is due to the fact that higher PSVs reflect reduced renal perfusion and greater resistance in the blood vessels. Conversely, a higher EDV usually correlates with a higher GFR [20].

The reduction in the PSV was also related to the UACR and eGFR, which are established markers of kidney function. An increase in the UACR is a sign of early kidney damage and is typically associated with a decline in the eGFR, which measures the glomerular filtration rate and reflects renal function [21]. Some studies explain that microalbuminuria is associated with high PSV [20].

The increase in the EDV after the dendritic cell therapy is also noteworthy. EDV is a measure of renal perfusion and is positively correlated with the glomerular filtration rate (GFR), a key marker of kidney function. Research indicates that a decrease in EDV will lead to increased vascular resistance, impaired perfusion, and reduced GFR [22]. In kidney disease, this decrease in the EDV will increase resistance in blood vessels and cause impaired perfusion and a decreased GFR [23]. Decreased EDV will also lead to increased albuminuria through a decreased GFR [24]. Some studies also explain the existence of an immune response, namely inflammation, in patients with microalbuminuria and macroalbuminuria [25].

Regarding gender-specific differences, our results indicate that significant changes in PSV and EDV were observed in the female group, while the men did not show significant changes. This finding may be explained by differences in immune responses between the genders, with women generally exhibiting stronger immune responses, possibly due to the influence of estrogen, which can enhance antibody production [26]. This finding indicates a potential gender-specific response and the need for further investigation in larger cohorts.

The differences in renal physiology and immune response based on age are the basis for the grouping in this study. This is in line with research conducted by Costagliola et al., which showed differences in immune responses based on age [27]. Research conducted by Weinstein et al. shows that there are changes in renal blood flow with age [28]. However, in this study, in both the age groups of under 60 and over 60 years, there were no significant changes in PSV and EDV.

We also explored the pathophysiological mechanisms of microalbuminuria and macroalbuminuria. Microalbuminuria is an early marker of kidney damage, whereas macroalbuminuria often indicates significant structural changes in the glomerulus [29,30]. In the microalbuminuria group, the PSV was significantly reduced after the dendritic cell therapy, whereas the EDV showed an increase, though not statistically significant. These findings suggest that early-stage DKD may respond more effectively to DC therapy, whereas advanced stages like macroalbuminuria, which involve substantial glomerular damage, may require more intensive treatment or longer follow-up periods to observe improvements in renal hemodynamics.

Linear regression analysis showed a reduction in the influence of MMP-9 on TGF-β after dendritic cell therapy, although this result approached statistical significance (*p* = 0.066). The difference between the Spearman test (showing an association between TGF-β and MMP-9) and the linear regression findings may be attributed to the small sample size, which limited the statistical power. Previous research has highlighted the interaction between TGF-β and MMP-9, showing that TGF-β activates MMP-9, which in turn promotes fibrogenesis [31]. Research conducted by Gu et al. showed that inhibition of TGF-β can reduce MMP-9 [32]. Muscella et al. stated that TGFβ activates cell migration through MMP 2 and MMP-9 [33]. In this study, there was a decrease in the influence of MMP-9 on TGF-β after the administration of the autologous dendritic cells. The decrease in the TGF-β was expected to reduce fibrosis in the kidney. TGF-β activates Smad2 and Smad 3 and then interacts with the transcription factors involved in fibrogenesis [34,35].

## 5. Conclusions

Autologous dendritic cell administration can positively impact renal hemodynamics in patients with DKD. Significant changes were observed in PSV and EDV, with improvements indicating enhanced renal blood flow. Notably, autologous dendritic cells were effective in reducing inflammation and fibrosis markers, particularly in the microalbuminuria group, suggesting a potential therapeutic benefit. Although no significant changes were seen in the macroalbuminuria group, longer treatment and follow-up may be required for patients with more advanced kidney damage. While the evidence for fibrosis reduction is promising, further studies with larger cohorts and more direct measures of fibrosis are required to confirm the therapeutic potential of DC therapy for DKD.

## Figures and Tables

**Table 1:** Characteristics of research subjects.

		Count	Table N %
Gender	Women	16	55.2%
	Men	13	44.8%
Age	<60	9	31.0%
	≥60	20	69.0%
BMI	Underweight	2	6.9%
	Normal weight	6	20.7%
	Overweight	0	0.0%
	Obesity I	13	44.8%
	Obesity II	8	27.6%
Hypertension	No	1	3.4%
	Yes	28	96.6%
Stroke	No	24	82.8%
	Infarction	5	17.2%
	Hemorrhagic	0	0.0%
Heart disease	No	19	65.5%
	Yes	10	34.5%
Retinopathy	No	25	86.2%
	Yes	4	13.8%
Neuropathy	No	13	44.8%
	Yes	16	55.2%
Biguanid	No	20	69.0%
	Yes	9	31.0%
Thiazolidinedione	No	29	100.0%
	Yes	0	0.0%
Glinid	No	29	100.0%
	Yes	0	0.0%
α glucosidase inhibitor	No	26	89.7%
	Yes	3	10.3%
Insulin	No	9	31.0%
	Yes	20	69.0%
Gliptin	No	23	79.3%
	Yes	6	20.7%
SGLT2	No	26	89.7%
	Yes	3	10.3%
Sulphonylurea	No	17	58.6%
	Yes	12	41.4%
Central alpha agonist	No	29	100.0%
	Yes	0	0.0%
Diuretics	No	27	93.1%
	HCT	2	6.9%
	Spironolacton	0	0.0%
Alpha blockers	No	28	96.6%
	Yes	1	3.4%
CCB	No	9	31.0%
	Dihydropyridine	15	51.7%
	Non-Dihydropyridine	5	17.2%
	DHP and non-DHP	0	0.0%
β blockers	No	21	72.4%
	Yes	8	27.6%
ARB	No	8	27.6%
	Yes	21	72.4%

Abbreviations: HCT = Hydrochlorothiazide, CCB = Calcium Channel Blocker, ARB = angiotensin II receptor blocker.

**Table 2:** Results of PSV and EDV analysis before and after autologous dendritic cell administration.

	Before (cm/s)	After (cm/s)	*p*-Value
PSV (Median ± IQR)	47.1 ± 23.87	27.85 ± 20.53	0.044
EDV (Median ± IQR)	13 ± 5.32	15.7 ± 12.55	0.039

Abbreviations: PSV = Peak Systolic Velocity, EDV = End-Diastolic Velocity, IQR = Interquartile Range.

**Table 3:** PSV by sex, age, and UACR before and after autologous dendritic cell administration.

		PSV Before (cm/s)	PSV After (cm/s)	*p*-Value
Gender *	Men	47.1 ±23.3	27.05 ±42.38	0.422
	Women	51.65 ±24.8	31.72 ±18.31	0.03
Age ^† ^	<60	52.56 ±18.41	42.32 ±24.80	0.225
	≥60	47.02 ±24.97	29 ±20.43	0.121
UACR *	Microalbuminuria	54.6 ±23.46	27.65 ±16.74	0.011
	Macroalbuminuria	47.05 ±32.3	35.7 ±32.28	0.834

Abbreviations: PSV = Peak Systolic Velocity, IQR = Interquartile Range, SD = Standard Deviation, UACR = Urinary Albumin-to-Creatinine Ratio. * Median ± IQR. ^†^ Mean ± SD.

**Table 4:** EDV by sex, age, and UACR before and after autologous dendritic cell administration.

		EDV Before (cm/s)	EDV After (cm/s)	*p*-Value
Gender *	Men	12.55 ±6.97	15.7 ±21.9	0.249
	Women	13.27 ±6.8	15.04 ±11.08	0.044
Age ^†^	<60	15.53 ±6.10	23.03 ±14.93	0.137
	≥60	4.11 ±6.08	12.64 ±11.08	0.126
UACR *	Microalbuminuria	13.8 ±5.36	14.19 ±11.18	0.234
	Macroalbuminuria	11.15 ±6.28	16.4 ±17.75	0.087

Abbreviations: EDV = End-Diastolic Velocity, IQR = Interquartile Range, SD = Standard Deviation, UACR = Urinary Albumin-to-Creatinine Ratio. * Median ± IQR. ^†^ Mean ± SD.

**Table 5:** Linear regression testing of TGF-β and MMP-9 before and after autologous dendritic cell administration.

Variables	Coefficient (β)	*p*-Value
MMP-9 Before	13.112	0.058
Dependent Variable TGF-β Before		
MMP-9 After	7.622	0.066
Dependent Variable TGF-β After		

Abbreviations: MMP-9 = Matrix Metalloproteinase-9, TGF-β = Transforming Growth Factor Beta.

**Table 6:** Analysis of the relationship between variables.

Variables	MMP-9 (r, *p*)	PSV (r, *p*)	EDV (r, *p*)
TGF-β	0.413, 0.001 **	−0.101, 0.452	−0.071, 0.598
MMP-9	-	−0.015, 0.909	−0.048, 0.721
PSV	−0.015, 0.909	-	0.675, 0.000 **
EDV	−0.048, 0.721	0.675, 0.000 **	-

Abbreviations: MMP-9 = Matrix Metalloproteinase-9, TGF-β = Transforming Growth Factor Beta, PSV = Peak Systolic Velocity, EDV = End-Diastolic Velocity. r = correlation coefficient. Note: ** *p* < 0.01.

## Data Availability

The data are unavailable due to privacy or ethical restrictions.

## Abbreviations

The following abbreviations are used in this manuscript:

DKD	Diabetic kidney disease
ESRD	End-Stage Renal Disease
PSV	Peak Systolic Velocity
EDV	End-Diastolic Velocity
MMP-9	Matrix Metalloproteinase-9
TGF-β	Transforming Growth Factor Beta
ELISA	Enzyme-Linked Immunosorbent Assay
GM-CSF	Granulocyte Macrophage Colony Stimulating Factor
IL-4	Interleukin-4
CTCAE	Common Terminology Criteria for Adverse Events
eGFR	Estimated Glomerular Filtration Rate
UACR	Urinary Albumin-to-Creatinine Ratio
BMI	Body Mass Index
HCT	Hydrochlorothiazide
CCB	Calcium Channel Blocker
ARB	Angiotensin II receptor blocker
